# SIENA Score and CVP/PCWP Predict Mid-Term Prognosis After LVAD Implantation: A Single-Center Study

**DOI:** 10.3390/jcdd13060273

**Published:** 2026-06-16

**Authors:** Giulia Elena Mandoli, Maria Barilli, Maria Concetta Pastore, Silvia Foli, Nicolò Ghionzoli, Federico Landra, Marta Focardi, Enrico Emilio Diviggiano, Flavio D’Ascenzi, Luna Cavigli, Sonia Bernazzali, Massimo Maccherini, Serafina Valente, Matteo Cameli

**Affiliations:** 1 Department of Medical Biotechnologies, Division of Cardiology, University of Siena, Policlinico “Le Scotte”, 53100 Siena, Italy; barillimaria@gmail.com (M.B.); pastore2411@gmail.com (M.C.P.); silvia.foli@student.unisi.it (S.F.); nicologhionzoli@gmail.com (N.G.); f.landra@student.unisi.it (F.L.); focardim@hotmail.com (M.F.); e.diviggiano@student.unisi.it (E.E.D.); flavio.dascenzi@unisi.it (F.D.); serafina.valente@unisi.it (S.V.); matteo.cameli@yahoo.com (M.C.); 2 Cardiac Surgery Unit, Cardio-Thoracic and Vascular Department, University of Siena, Policlinico “Le Scotte”, 53100 Siena, Italy; s.bernazzali@gmail.com (S.B.); maccherini2@unisi.it (M.M.)

**Keywords:** left ventricular assist device, right ventricular failure, echocardiography, right heart catheterization

## Abstract

(1) Background: Left ventricular assist device (LVAD) implantation is a valuable alternative as a bridge to transplant but also as a destination therapy in ineligible patients. Right ventricular failure (RVF) is a major cause of short- and long-term mortality post-LVAD. We aimed to validate echocardiographic and hemodynamic parameters predictive of RVF and adverse outcomes post-LVAD; (2) Methods: We screened a population of patients with end-stage heart failure selected for LVAD implantation according to SIENA protocol and standard international indications, including right heart catheterization (RHC). Individuals were followed up for 1 year with different time points for the development of RVF (primary endpoint) or mortality and hospitalization (secondary endpoint); (3) Results: The population included 29 patients with a mean age of 63 ± 7 years with a mean ejection fraction of 23 ± 4%, mostly due to ischemic etiology. All the patients had a SIENA protocol score of 0–1 before LVAD, and none met the primary endpoint. Regarding the secondary endpoint, among all the tested clinical, laboratory, echo, and RHC indices, only a central venous pressure/wedge pressure (CVP/PCWP) ratio > 0.63 was significantly associated with adverse outcomes (ß = 2.99, *p* = 0.026); (4) Conclusions: Excluding a pre-implantation RV dysfunction according to SIENA protocol significantly reduces the risk of post-LVAD RVF. The CVP/PCWP ratio may be an additional prognostic marker for mortality and rehospitalization in LVAD patients.

## 1. Introduction

Patients with advanced heart failure (AdHF) comprise approximately 11% of the total heart failure population, with some variability across heart failure phenotypes. These patients face a significant mortality rate, ranging from 25% to 75% within the first year [1]. Additionally, their quality of life is severely compromised as they are classified in the highest NYHA functional classes (III or IV) and experience persistent and severe symptoms [2]. Heart transplantation (HTx) is the primary treatment when conventional medical therapy is inadequate and in the absence of contraindications. However, due to limited organ availability, an average of 10–15% of patients on the transplant waiting list die each year in European countries [3].

For patients with isolated left ventricular (LV) failure, long-term mechanical circulatory support (MCS) is often indicated to prolong survival and enhance quality of life. Indications for MCS vary, including bridge to HTx, bridge to candidacy (e.g., cases of pulmonary hypertension or recent malignancy), or destination therapy (DT), especially for those patients with AdHF and advanced age. The MOMENTUM 3 trial showed a five-year survival rate of 58.4% in the United States, with similar rates reported in Europe [4]. Right ventricular failure (RVF) remains a leading cause of mortality over time [5]. It occurs with an estimated prevalence of 8–10% in patients with continuous-flow LVADs [6] and an incidence of approximately 25% [7].

**Figure 1:**
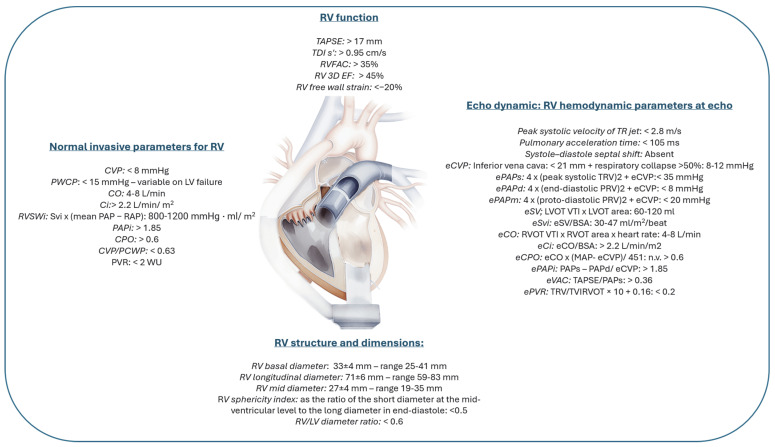
Invasive and non-invasive parameters for RV geometry and function are described in the normal range. All the parameters preceded by an “e” are echo derived [5,11,12,14,15,16]. BSA: body surface area; Ci: cardiac index; CO: cardiac output; CPO: cardiac power output; CVP: central venous pressure; LVOT VTI: left ventricular outflow tract velocity-time integral; PAPi: pulmonary artery pulsatility index; PVR: pulmonary vascular resistance; PCWP: pulmonary capillary wedge pressure; RV 3D EF: right ventricular 3D ejection fraction; RVFAC: right ventricular fractional area change; RVSWi: right ventricular stroke work index; SV: stroke volume; TAPSE: tricuspid annular plane systolic excursion; TDI: tissular doppler imaging; eVAC: ventricular–arterial coupling.

The latest consensus document on echocardiographic assessment of LVAD candidates and recipients highlights critical aspects of RV evaluation through a multiparametric approach [5]. Many AdHF patients present challenges in clinical assessment due to the variability of phenotypes, which are often difficult to categorize. Medical therapy, MCS, mechanical ventilation, arrhythmias, and rapidly changing clinical statuses can substantially affect imaging evaluations, necessitating a multiparametric approach for each case. Transthoracic echocardiography (TTE) plays a pivotal role in pre-implantation selection, offering information on right and left ventricular dimensions and function, valvular anatomy, intracavitary pressures, and hemodynamic parameters, including cardiac output, pulmonary resistance, ventricular interdependence, and ventricular–arterial coupling [8,9]. In RV assessment, it is crucial to evaluate adaptation to elevated afterload due to left-sided heart disease, characterized by initial contractility elevation, compensatory hypertrophy, and eventual dilation [10]. However, this adaptation may be absent in acute cases with abrupt afterload increases and pressure elevation [11]. Key TTE parameters include TAPSE, lateral annulus TDI, fractional area change, sphericity index, RV/LV diameter ratio, PAPs, inferior vena cava diameter with respiratory variation, global longitudinal RV strain, systole-diastole septal shift, TAPSE/PAPs, and peak systolic velocity of TR/VTI RVOT (Figure 1). Due to RV’s complex structure, 3D echocardiography and transesophageal echocardiography (TOE) should be incorporated into pre-implantation evaluations [5,12]. All pre-LVAD patients should undergo hemodynamic assessment through right heart catheterization or continuous Swan–Ganz catheter monitoring. These invasive measures provide a comprehensive RV functional profile, including stroke work, pressure evaluation, RV-pulmonary circulation coupling, pulmonary artery resistance, and right-to-left filling pressure [11,13] (Figure 1).

The purpose of this study was to identify the invasive and non-invasive predictive parameters of RVF development after LVAD implantation with a follow-up of 12 months. For prognostic and therapeutic evaluation, we used the “SIENA protocol,” a validated tool incorporating four RV function and geometry indices: Tricuspid Annular Plane Systolic Excursion (TAPSE), Right Ventricular Fractional Area Change (RVFAC), Right Ventricular Sphericity Index (RVSI), and longitudinal strain of the right ventricular free wall (Free-Wall RVLS) using speckle tracking [14].

## 2. Materials and Methods

This single-center study collected data on patients with AdHF, as defined by the Heart Failure Association–European Society of Cardiology (HFA-ESC) criteria, who met eligibility for LVAD implantation and had the parameters available to calculate the SIENA score. Patients lacking informed consent, complete pre-operative echocardiographic examination, or with absent follow-up data were excluded.

The primary endpoint was the identification of RVF following LVAD implantation, defined by persistent signs or symptoms of RV failure, elevated central venous pressure (CVP ≥ 18 mmHg) with reduced cardiac index (<2.3 L/min/m^2^) in the absence of elevated pulmonary capillary wedge pressure (PCWP < 18 mmHg), or the presence of cardiac tamponade, ventricular arrhythmias, pneumothorax, the need for right ventricular assist device (RVAD) implantation, or prolonged use of inhaled nitric oxide (≥48 h) or inotropic therapy (≥14 days).

Secondary endpoints included individual and composite outcomes encompassing all-cause mortality, cardiovascular mortality (including deaths due to acute heart failure, acute myocardial infarction, ventricular tachycardia or fibrillation, sudden cardiac arrest, and stroke), and HF-related rehospitalization.

### 2.1. Baseline and Follow-Up Data

Baseline data collection included comprehensive medical history, cardiovascular risk factors, medical treatments, the origin and type of cardiomyopathy (ischemic or non-ischemic), myocardial infarction subtype, prior cardiac surgeries, paroxysmal or chronic atrial fibrillation, and the presence of devices such as pacemakers or implantable cardioverter defibrillators. Functional status and quality of life were assessed using the Minnesota Living with Heart Failure Questionnaire, Interagency Registry for Mechanically Assisted Circulatory Support (INTERMACS) classification, and the six-minute walk test. Vital signs, HF signs and symptoms, medical therapies, and blood analyses were documented. Post-LVAD and length of ICU stay were recorded, and outpatient follow-ups were conducted at 1, 3, 6, 9, and 12 months, with the same data collected as at baseline. All patients signed informed consent.

### 2.2. Echocardiographic Exam

Echocardiographic assessments (both standard and advanced, including 3D and speckle-tracking echocardiography) were performed on high-quality machines using a 2.5 MHz transducer (GE Healthcare, Milwaukee, WI, USA). Transthoracic echocardiography followed AHA and ESC-EACVI guidelines [15], and speckle-tracking analysis was completed offline using semi-automated 2D software (V:204) (EchoPac, GE, Milwaukee, Madison, WI, USA). All patients received a score according to the SIENA protocol.

### 2.3. Right Heart Catheterization

Right heart catheterization (RHC) was conducted within four weeks of echocardiography, prior to LVAD implantation, with measurements taken at end-expiration following standardized protocols [16]. Hemodynamic parameters included mean, systolic, and diastolic pulmonary artery pressures (mPAP, sPAP, dPAP), right atrial pressure (RAP), and average PCWP. Calculated parameters included pulmonary vascular resistance, the RAP/PCWP ratio, pulmonary artery pulsatility index (PAPi), and right ventricular stroke work index (RVSWI). Cardiac output and cardiac index were measured via thermodilution and the indirect Fick method.

### 2.4. Statistical Analysis

Potential outcome predictors were analyzed using univariate and multivariate analyses, focusing on right ventricular failure, cardiovascular and all-cause mortality, rehospitalization, and bleeding events post-LVAD implantation. Data analysis was performed with IBM SPSS 25.0 (1989–2017, LEAD Technologies Inc., Charlotte, NC, USA). The Kolmogorov-Smirnov test assessed the distribution of continuous variables: normally distributed variables are presented as mean ± standard deviation, non-normally distributed variables as median (interquartile range), and categorical variables as frequency (percentage). Cox univariate analysis was also conducted. A *p* value < 0.05 was considered statistically significant.

## 3. Results

Twenty-nine patients were selected at our center from May 2013 to February 2022. In this cohort, 93% were male, with a mean age of 63 ± 7 years and an average BMI of 26.38 ± 5.67. Cardiovascular risk factors, including hypertension, diabetes, dyslipidaemia, and smoking, were present in 58%, 47%, 63%, and 65% of patients, respectively. Coronary artery disease (CAD) was reported in 29% of cases. The whole population of patients was classified as NYHA class III-IV, while INTERMACS profiles 3, 4, and 5 included 40%, 46%, and 14% of the population, respectively. Ischemic etiology accounted for 58% of the heart failure cases, and 44% of patients had cardiac resynchronization therapy (CRT) devices.

After selecting the patients according to the SIENA protocol and international guidelines, none of the patients developed RVF.

**Figure 2:**
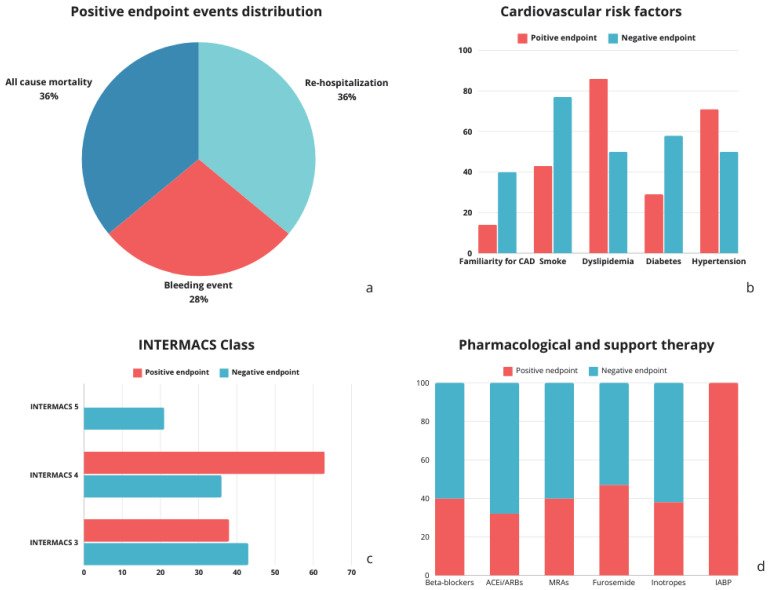
(**a**) Adverse effects in the positive endpoint group; (**b**) Cardiovascular risk factors in the negative and positive endpoint groups; (**c**) INTERMACS Class distribution in the negative and positive endpoint groups; (**d**) Medical therapy distribution in the negative and positive endpoint groups. ACEi/ARB, angiotensin converting enzyme inhibitor/angiotensin II receptor blockers; BB, beta blockers; CAD: coronary artery disease; IABP, intra-aortic balloon pump; MRAs, mineralocorticoid receptor antagonists.

The cohort was then divided into two groups based on the occurrence of the study’s secondary composite endpoint. The “no endpoint” group included 18 patients, while 11 patients experienced adverse cardiovascular outcomes. Of these, 4 required rehospitalization, 3 experienced bleeding events, and 4 died from all causes (Figure 2a). An analysis of risk factors associated with adverse cardiovascular events revealed no significant differences in hypertension, diabetes, dyslipidaemia, smoking, or family history of CAD when comparing patients with and without adverse events, as shown in Figure 2b. Differences between groups by INTERMACS profile are displayed in Figure 2c.

Anthropometric and baseline characteristics, stratified by endpoint occurrence, are summarized in Table 1. No statistically significant differences were observed between the groups. Blood chemistry data, evaluated pre- and post-LVAD implantation, also showed no statistically significant differences between the two groups (Table 2). Regarding pharmacologic treatments, there were no statistically significant differences based on endpoint occurrence, despite variations in primary medications (Figure 2d and Table 3). Data from standard and advanced echocardiographic evaluations, including speckle-tracking echocardiography, are listed in Table 4. Cardiac catheterization data are summarized in Table 5, with results provided for the total population and stratified by endpoint occurrence. No significant differences in hemodynamic parameters were observed between patients with and without adverse events. Univariate analysis of composite event predictors, conducted using Cox regression with significance set at *p* < 0.05, revealed a statistically significant correlation only for the CVP/PCWP ratio (*p* = 0.026), as shown in Table 6.

## 4. Discussion

Long-term MCS, such as LVADs, was first developed in the 1960s as a response to the global shortage of donor organs for patients with AdHF. Initially designed to support the heart and circulation during prolonged waiting periods for transplantation, the application of LVADs has broadened in recent years to include patients who are ineligible for transplant due to factors such as advanced age or relative contraindications. The American Heart Association (AHA) and European Society of Cardiology (ESC) guidelines recommend long-term mechanical support as a Class IIa indication for advanced heart failure patients, preferably those with INTERMACS Class 3–5 profiles [2,17]. Selecting optimal LVAD candidates is challenging due to the diversity of patient phenotypes. One of the primary considerations before implantation is RV function, as RV failure significantly worsens prognosis [6]. The MOMENTUM 3 trial reported that the cumulative incidence of RVF in HeartMate III recipients was approximately 35% two years post-implant. Patients without RVF had a mortality rate of 53% at one year compared to 71% in patients with RVF, and 45% versus 58% at two years [18]. The prognosis and quality of life for patients following long-term MCS implantation depend significantly on their pre-surgical clinical and instrumental profile. Thus, a systematic assessment of patients with end-stage heart failure, both before and after implantation, may help identify optimal clinical, laboratory, invasive, and echocardiographic parameters predictive of RVF post-LVAD implantation. Prior studies identified the factors associated with RVF risk, such as inotropic support requirement, high INTERMACS classification, and biomarkers [19,20]. Hemodynamic parameters obtained via right heart catheterization are closely associated with RVF prediction, including increased pulmonary vascular resistance, the central venous pressure (CVP) to pulmonary capillary wedge pressure (PCWP) ratio [21], pulmonary artery pulsatility index (PAPi), and RVSWi [22]. Additionally, advanced echocardiographic parameters, such as right ventricular longitudinal deformation assessed by speckle-tracking echocardiography (STE), are associated with invasive RV functional measures [23] and have shown prognostic value in patients undergoing LVAD implantation [24,25]. Despite these advances, existing scores demonstrate only moderate predictive power for these patients [26].

In our single-center study, we conducted a rigorous pre-implant analysis of invasive and non-invasive data (including echocardiographic SIENA protocol) and followed patients for 12 months post-implant. RVF development was closely monitored as the primary endpoint. During the 12-month follow-up, none of our patients developed RVF. For secondary outcomes, including all-cause mortality, hemorrhagic events, and rehospitalizations, the only statistically significant predictor was the CVP/PCWP ratio (OR: 19.896; *p* = 0.026), underscoring the prognostic importance of cardiac catheterization prior to LVAD implantation. Consistent with International Society for Heart and Lung Transplantation guidelines, invasive assessments should be conducted for all potential candidates [13]. In acute cases, the placement of a Swan–Ganz catheter allows for detailed evaluation of the patient’s pulmonary status, optimizing medical and congestive management before implantation and determining whether elevated pulmonary vascular resistance is reversible with vasodilators. This assessment also aids in evaluating RV function and cardiac output, which are essential for both pre- and post-operative management [27]. Among the 11 patients who experienced adverse events, 36% required rehospitalization, 27% had hemorrhagic events, and 36% died. Hemorrhagic complications are common in this patient population due to the delicate balance between pro-thrombotic and hemorrhagic factors and the requirement for anticoagulation therapy [28]. Rehospitalizations were also frequent, often driven by infection risks and probably due to the advanced age of the patient population, with a mean age of 63 ± 7.4 years. Given the impact of RVF on post-operative morbidity and mortality, our follow-up aimed to identify predictors of adverse events from clinical, anthropometric, biochemical, echocardiographic, and catheterization data. Patient selection for LVAD at our center involves a comprehensive assessment to ensure that candidates will benefit from long-term support [14]. As detailed by Morgan et al., a careful selection process, emphasizing parameters such as CVP, right ventricular stroke work index (RVSWI), echocardiographic RV contractility, and clinical symptoms of RV failure, is essential for reducing RVF [29]. Several studies, including those by Fukamani et al., Karavana et al., and Matthews et al., have identified RVSWI, pre-operative pulmonary artery pressure (PAP), bilirubin levels, and RV contractility indices as significant predictors of RVF [30,31,32]. For instance, Fukamani et al. noted that low pre-operative PAP and RVSWI, indicative of poor RV contractility, increased the risk of RVF in HeartMate implantation patients [30]. Similarly, Kormos et al. identified a CVP/PCWP ratio >0.63 (OR: 2.3, *p* = 0.009), ventilatory support requirement, and elevated blood urea nitrogen (BUN) as independent predictors of RVF post-LVAD [33]. In our population, none of the patients had pre-operative bilirubin or PAP levels similar to those associated with RVF in previous studies. From the European Registry of Patients with Mechanical Circulatory Support (EUROMACS) registry, predictors of early mortality in LVAD patients include elevated creatinine, bilirubin, low hemoglobin, and advanced INTERMACS profiles [21]. In our cohort, however, factors such as creatinine, hemoglobin, and INTERMACS profile 3 did not reach statistical significance, likely due to the different mortality causes in our study compared to multi-organ failure and sepsis in the EUROMACS population. Additionally, the recent literature underscores elevated NT-proBNP as a potential predictor of RVF and adverse events in LVAD patients, though our analysis found no significant association with rehospitalizations [34]. Risk scores, such as the HeartMate Risk Score (HMRS) [35] and others focused on NT-proBNP, CVP, and BUN, offer valuable insights into early mortality and RVF risks, although many of these parameters were not statistically significant in our cohort, possibly due to sample size and comorbidity variability. Pharmacologic treatment studies, such as those by Brinkley et al., highlight the protective effects of ACE inhibitors and mineralocorticoid receptor antagonists (MRAs) against cardiovascular mortality and gastrointestinal bleeding [36], although these therapies were not statistically significant in our cohort. Furthermore, studies from the University of Siena and other centers highlight the prognostic value of advanced RV function parameters, such as PAPi and Free-Wall RVLS, with Free-Wall RVLS emerging as a key predictor of RVF in several cohorts [22,37,38]. However, in our study, this measure did not show a similar association, consistent with the absence of RVF development in our population.

Our secondary objective of identifying the predictors of all-cause mortality, bleeding events, and rehospitalizations found the CVP/PCWP ratio to be the only hemodynamic parameter with statistical significance (*p* = 0.026). This aligns with EUROMACS findings linking elevated CVP/PCWP ratios and other hemodynamic indicators to early mortality risk [21]. The relationship between CVP and PCWP reflects an imbalance between right and left filling pressures. An elevation of CVP disproportionate to the elevation of PCWP, in cases of CVP elevated above normal, is representative of RV dysfunction, as in normal conditions CVP is significantly lower than PCWP [39]. Sabashnikov et al. also demonstrated that increased CVP and advanced age are critical post-LVAD mortality predictors [40]. Although age > 45 years and CVP > 18 mmHg were significant in their study, Adamson et al. noted no outcome difference based on age > 70 years, challenging age as an absolute LVAD contraindication [41]. Elevated CVP, however, remains a robust predictor of post-operative RVF and adverse outcomes, as confirmed by Mehra et al., who associated a CVP/PCWP ratio >0.6 with increased mortality [42].

Overall, our findings underscore the prognostic significance of pre-operative hemodynamic assessments, particularly the CVP/PCWP ratio, in optimizing outcomes and mitigating the risk of RVF post-LVAD implantation. These insights support the critical role of both invasive and non-invasive measures in selecting and managing LVAD candidates, with particular attention to RV function and hemodynamic stability as predictors of post-operative success.

### Limitations

The limitations of our study align with those frequently observed in the existing literature, as most case series on post-LVAD right ventricular failure (RVF) involve relatively small sample sizes. The lack of a consistent, universally accepted definition of RVF limits standardization and complicates the assessment of outcomes in this patient population. Furthermore, the small sample size heightens the risk of confounding variables and reduces the generalizability of findings to a broader population. As an observational, monocentric study, our results should be interpreted cautiously due to limited statistical power, an increased risk of Type II errors, and a greater likelihood of selection bias. Future research with larger, prospective studies is essential to validate these conclusions.

## 5. Conclusions

This study highlights the importance of a comprehensive pre-operative assessment for patients with advanced heart failure who are candidates for long-term MCS through LVADs. In particular, a systematic echocardiographic assessment of potential candidates increases the possibility of avoiding RVF after implantation. Moreover, our findings underscore that the CVP/PCWP ratio can be a significant predictor of adverse outcomes, including rehospitalization, hemorrhagic events, and mortality, aligning with other studies that emphasize the need for precise hemodynamic evaluation prior to LVAD implantation. By utilizing a combination of invasive and non-invasive parameters, healthcare providers can optimize patient selection and improve post-operative outcomes, specifically by focusing on RV function. The study underscores the role of individualized risk assessments and the potential of predictive scores, such as the CVP/PCWP ratio, in informing treatment strategies that enhance the longevity and quality of life for LVAD patients.

## Figures and Tables

**Table 1:** Medical history and anthropometrical data of the population.

Variable	Population (*n* = 29)	Negative Endpoint Group (*n* = 18)	Positive Endpoint Group (*n* = 11)	*p* Value
Male gender (%)	93	100	82	0.135
Age (years)	62 ± 7.4	62 ± 8.3	63 ± 5.9	0.926
BMI (kg/m^2^)	26 ± 5.7	26 ± 6.4	26 ± 4.6	0.927
Hypertension (%)	58	50	71	0.633
Diabetes (%)	47	58	29	0.350
Dyslipidaemia (%)	63	50	86	0.173
Smoker/ex-smoker (%)	65	77	43	0.174
CAD Familiarity (%)	29	40	14	0.338
Ischemic etiology (%)	58	59	56	0.990
CRT (%)	44	50	33	0.683
NYHA III-IV (%)	100	-	-	0.177
INTERMACS 3 (%)	40	43	38	0.991
INTERMACS 4 (%)	46	36	63	0.378
INTERMACS 5 (%)	14	21	0	0.273
SBP (mmHg)	103 ± 15.2	103 ± 15.3	104 ± 15.8	0.947
CF (bpm)	73 ± 11.5	74 ± 13.6	70 ± 5.5	0.382

BMI, body mass index; CAD, coronary artery disease; CF, cardiac frequency; CRT, cardiac resynchronization therapy; SBP, systolic blood pressure.

**Table 2:** Biochemistry data of the population.

Variable	Population (*n* = 29)	Negative Endpoint Group (*n* = 18)	Positive Endpoint Group (*n* = 11)	*p* Value
Hb (g/dL)	13.0 ± 1.2	13.7 ± 1.2	12.6 ± 2.3	0.765
PLT (× 10^3^) (cell/mm^3^)	187 ± 45.9	187 ± 28.3	187 ± 71.1	0.987
NT-proBNP (pg/mL)	3308 ± 2174.5	3336 ± 2127.4	3235 ± 2551.3	0.939
Glycaemia (mg/dL)	110 ± 47.5	100 ± 27.1	129 ± 71.2	0.210
Creatinine (mg/dL)	1.46 ± 0.4	1.36 ± 0.4	1.68 ± 0.5	0.107
eGFR	56 ± 13.5	63 ± 13.5	44 ± 7.5	0.066
CRP (mg/dL)	1.57 ± 2.0	1.95 ± 2.3	0.92 ± 0.9	0.281
Total Bilirubin (mg/dL)	0.77 ± 0.3	0.76 ± 0.3	0.78 ± 0.4	0.941

CRP, C reactive protein; eGFR, estimated glomerular filtration rate; Hb, hemoglobin; NT-proBNP, N-terminal prohormone of brain natriuretic peptide; PLT, platelets.

**Table 3:** Medical therapy of the population.

Variable	Population (*n* = 29)	Negative Endpoint Group (*n* = 18)	Positive Endpoint Group (*n* = 11)	*p* Value
BB (%)	82	92	60	0.191
ACEi/ARB (%)	35	42	20	0.600
MRA (%)	82	92	60	0.191
Furosemide (%)	88	92	80	0.515
Inotropes (%)	29	33	20	0.990
IABP (%)	6	0	17	0.333

ACEi/ARB, angiotensin converting enzyme inhibitor/angiotensin II receptor blockers; BB, beta blockers; CAD, coronary artery disease; IABP, intra-aortic balloon pump; MRAs, mineralocorticoid receptor antagonists.

**Table 4:** Main standard and advanced echocardiographic parameters with speckle-tracking technique.

Variable	Population (*n* = 29)	Negative Endpoint Group (*n* = 18)	Positive Endpoint Group (*n* = 11)	*p* Value
LAVi (ml/m^2^)	67 (56–72)	67 (59–73)	65 (45–69)	0.329
LVEDD (mm)	74 ± 8.7	74 ± 7.7	73 ± 10.5	0.717
LVEDV (mL)	279 ± 84.4	287 ± 87.7	266± 82.4	0.575
IVS (mm)	9.7 ± 1.9	9.5 ± 1.6	10 ± 2.5	0.507
LVEF (%)	23 ± 4.5	23 ± 3.7	23 ± 5.9	0.645
E/e’	18 ± 11.0	18 ± 11.7	18.3 ± 10.3	0.942
RV EDDm (mm)	34 ± 2.5	35 ± 3.5	33 ± 1.5	0.387
RVSI	0.45 ± 0.1	0.45 ± 0.1	0.45 ± 0.1	0.946
TAPSE (mm)	17 ± 3.9	18 ± 3.9	16 ± 4.1	0.468
S’-TDI (m/s)	0.11 ± 0.03	0,11 ± 0.03	0.10 ± 0.03	0.304
RVFAC (%)	36 ± 5.9	36 ± 6.1	37 ± 5.8	0.776
FW-RVLS (%)	−15 ± 5.1	−15 ± 3.4	−14 ± 7.7	0.819
PAPs (mmHg)	47 ± 12.9	46 ± 9.4	50 ± 17.5	0.462
LV-GLS (%)	−5 ± 1.3	−5 ± 1.1	−6 ± 1.6	0.306
LV-GLS 4Ch (%)	−5 ± 2	−4 ± 1.9	−6 ± 2.1	0.216
LV-GLS 2Ch (%)	−6 ± 2.4	−5 ± 2.5	−7 ± 2.2	0.276
LV-GLS 3Ch (%)	−5 ± 1.7	−4 ± 0.7	−6 ± 2.1	0.122
PALS (%)	6 ± 3.1	6 ± 3.0	7 ± 3.3	0.352

IVS, interventricular septum; LAVi, left atrium volume indexed; LVEDD, left ventricular end-diastolic diameter; LVEDV, left ventricular end-diastolic volume; LVEF, left ventricular ejection fraction; LVGLS, left ventricular global longitudinal strain; PALS, peak atrial longitudinal strain; PAPs, pulmonary artery pressure; RV EDD, right ventricular end-diastolic diameter; RVFAC, right ventricular fractional area change; FWRVLS, right ventricular free-wall longitudinal strain; TAPSE, tricuspid annular plane systolic excursion.

**Table 5:** Hemodynamic parameters data.

Variable	Population (*n* = 29)	Negative Endpoint Group (*n* = 18)	Positive Endpoint Group (*n* = 11)	*p* Value
CI (L/min/m^2^)	1.86 ± 0.4	1.85 ± 0.4	1.86 ± 0.4	0.965
PAPs (mmHg)	46 ± 16.3	45 ± 16.0	49 ± 17.0	0.450
PAPm (mmHg)	31 ± 10.9	30 ± 10.5	33 ± 11.9	0.507
CVP (mmHg)	7.6 ± 4.5	7.1 ± 4.1	8.4 ± 5.1	0.455
PCWP (mmHg)	20 ± 6.8	21 ± 6.9	20 ± 6.9	0.721
PVR (term) (WU)	3.6 ± 2.0	3.4 ± 1.8	3.4 ± 2.4	0.513
PVR indirect Fick(WU)	3.48 ± 2.1	3.4 ± 1.8	4 ± 2.5	0.420
CVP/PCWP	0.40 ± 0.2	0.35 ± 0.1	0.5 ± 0.3	0.165
PAPi	5.5 ± 5.1	4.5 ± 2.6	6.9 ± 7.5	0.227

CVP, central venous pressure; CI, cardiac index; PAP, pulmonary artery pressure; PCWP, pulmonary capillary wedge pressure; PVR, pulmonary vascular resistance.

**Table 6:** Predictors of the composite event with confidence intervals and *p* values.

Predictors Parameters	B	Exp(B) = HR	C.I.	*p* Value
INTERMACS 3	0.2	1.2	0.2–5.3	0.756
INTERMACS 4	−0.9	0.4	0.1–1.7	0.224
INTERMACS 5	3.3	26.6	0.1–91.5	0.430
BB	1.2	3.2	0.5–19.4	0.201
ACEi/ARB	0.9	2.4	0.3–21.4	0.436
MRA	1.3	3.7	0.6–22.8	0.159
Furosemide	0.5	1.6	0.2–14.4	0.671
Inotropes	0.6	1.8	0.1–15.8	0.610
IABP	−1.0	0.4	0.04–2.1	0.337
Hb	0.01	1.01	0.7–1.4	0.952
PLT	−0.001	0.1	1–1.02	0.886
Creatinine	1.4	4.1	0.7–25.4	0.126
eGFR	−0.04	0.1	0.9–1.02	0.177
NT-proBNP	0.001	1.0	0.1–1.0	0.959
LAVi	−0.01	0.1	0.9–1.02	0.445
LVEDD	−0.02	1.0	0.9–1.05	0.518
LVEDV	−0.003	0.1	1.0–1.0	0.463
LVEF	0.05	1.05	0.9–1.2	0.492
E/e’	0.1	1.09	1.0–1.2	0.064
RV EDDm	−0.03	1.0	0.9–1.06	0.529
RVSI	0.2	1.2	0.01–2	0.949
PAPs	0.03	1.03	1.0–1.08	0.290
TAPSE	−0.06	0.9	0.8–1.1	0.471
S’-TDI	−12	0.001	0.001–101	0.269
RVFAC	0.01	1.01	0.9–1.1	0.823
FW-RVLS	0.01	1.02	0.9–1.2	0.829
CI	0.1	1.1	0.3–4.1	0.894
PAPm	0.02	1.02	1.0–1.08	0.471
CVP	0.08	1.08	0.9–1.2	0.263
PCWP	−0.02	1.0	0.9–1.06	0.598
CVP/PCWP	3.0	19.9	1.4–275.4	0.026
PAPi	0.07	1.07	1.0–1.2	0.192

ACEi/ARB, angiotensin converting enzyme inhibitor/angiotensin II receptor blockers; BB, beta blockers; CI, cardiac index; CVP, central venous pressure; eGFR, estimated glomerular filtration rate; Hb, hemoglobin; IABP, intra-aortic balloon pump; LAVi, left atrium volume indexed; LVEDD, left ventricular end-diastolic diameter; LVEDV, left ventricular end-diastolic volume; LVEF, left ventricular ejection fraction; LVGLS, left ventricular global longitudinal strain; MRA, mineralocorticoid receptor antagonist; NT-proBNP, N-terminal prohormone of brain natriuretic peptide; PAPs, pulmonary artery pressure; PCWP, pulmonary capillary wedge pressure; PLT, platelet; RV EDD, right ventricular end-diastolic diameter; RVFAC, right ventricular fractional area change; FWRVLS, right ventricular free-wall longitudinal strain; TAPSE, tricuspid annular plane systolic excursion.

## Data Availability

The data presented in this study are available on request from the corresponding author. The data are not publicly available due to privacy and ethical restrictions.

## Abbreviations

The following abbreviations are used in this manuscript:

ACE	Multidisciplinary Digital Publishing Institute
AdHF	advanced heart failure
AHA	American Heart Association
BMI	body mass index
BUN	blood urea nitrogen
CAD	coronary artery disease
CRT	cardiac resynchronization therapy
CVP	central venous pressure
dPAP	diastolic pulmonary artery pressure
DT	destination therapy
ESC	European Society of Cardiology
EACVI	European Association of Cardiovascular Imaging
EUROMACS	European Registry of Patients with Mechanical Circulatory Support
Free-Wall RVLS	free-wall right ventricular longitudinal strain
HF	heart failure
HMRS	HeartMate Risk Score
HTx	heart transplantation
IBM	International Business Machines
ICU	intensive care unit
INTERMACS	Interagency Registry for Mechanically Assisted Circulatory Support
LV	left ventricle/left ventricular
LVAD	left ventricular assist device
MCS	mechanical circulatory support
mmHg	millimeters of mercury
MRA	mineralocorticoid receptor antagonist
mPAP	mean pulmonary artery pressure
NT-proBNP	N-terminal pro-B-type natriuretic peptide
NYHA	New York Heart Association
OR	odds ratio
PAP	pulmonary artery pressure
PAPi	pulmonary artery pulsatility index
PAPs	systolic pulmonary artery pressure
PCWP	pulmonary capillary wedge pressure
RHC	right heart catheterization
RV	right ventricle/right ventricular
RVAD	right ventricular assist device
RVF	right ventricular failure
RVFAC	right ventricular fractional area change
RVLS	right ventricular longitudinal strain
RVOT	right ventricular outflow tract
RVSI	right ventricular sphericity index
RVSWI	right ventricular stroke work index
SPSS	Statistical Package for the Social Sciences
STE	speckle-tracking echocardiography
TAPSE	tricuspid annular plane systolic excursion
TDI	tissue Doppler imaging
TOE	transesophageal echocardiography
TR	tricuspid regurgitation
TTE	transthoracic echocardiography
VTI	velocity-time integral
